# Reduction in Anti-Dengue Virus IgG Antibody Levels with the Use of a Larvicide for Vector Control in Rural Lao People’s Democratic Republic

**DOI:** 10.3390/tropicalmed8010020

**Published:** 2022-12-27

**Authors:** Pheophet Lamaningao, Seiji Kanda, Takaki Shimono, Mariko Kuroda, Somchit Inthavongsack, Thonelakhanh Xaypangna, Toshimasa Nishiyama

**Affiliations:** 1 Department of Hygiene and Public Health, Kansai Medical University, Osaka 573-1010, Japan; 2 Regenerative Research Center for Intractable Diseases, Kansai Medical University, Osaka 573-1010, Japan; 3 Station of Malariology, Parasitology, and Entomology Khammouane Provincial Health Department, Thakhek, Laos; 4 Khammouane Provincial Health Department, Thakhek, Laos

**Keywords:** *Aedes aegypti*, *Aedes albopictus*, dengue, larvicide, Lao PDR, pyriproxyfen, rural, SumiLarv^TM^ 2MR, NS1, ELISA

## Abstract

The Lao People’s Democratic Republic is an endemic area of dengue, with cases reported in urban and rural areas every year. In this study, we indirectly evaluated the efficacy of a larvicide (SumiLarv^TM^ 2MR discs) that was used for vector control against *Aedes* mosquitoes. Villages in a rural area of Lao PDR were selected as study areas, non-intervention and intervention villages. At the intervention village, the larvicide was used to treat refillable water containers for 27 months (October 2017 to February 2020), while at the non-intervention villages were no treatment. The serum samples of villagers from both villages were randomized to collect in the pre-intervention and in post-intervention periods. An enzyme-linked immunosorbent assay (ELISA) was used to examine anti-dengue virus (DENV) IgG antibody levels in serum samples. Recombinant DENV serotype 2 non-structural protein1 was used as an antigen for the ELISA, the optical density (OD) values were analyzed for comparison. The results showed that the OD values decreased significantly (*p* < 0.01) between the pre-intervention and post-intervention periods at the intervention site. The treatment of water storage containers in rural areas with SumiLarv^TM^ 2MR discs may help to protect residents from *Aedes* mosquito bites, and hence, reduce DENV infections.

## 1. Introduction

In the Lao People’s Democratic Republic (PDR), dengue cases are reported every year and large outbreaks also were reported from time to time [1]. The first reported cases of dengue in Lao PDR were 37 cases of dengue hemorrhagic fever reported in 1979, although none of these cases resulted in death. Large outbreaks subsequently occurred in 1985 (1774 cases, 15 deaths) and 1987 (9699 cases, 295 deaths). During the dengue outbreak in 1987, Khammouane Province reported 99 cases (2 deaths), and significant numbers of cases were reported in 6 other provinces (Vientiane Capital City, Vientiane Province, Champasak Province, Savannakhet Province, and LuangPrabang Province) [2]. Two decades later, dengue was reported to be endemic in every province in the country [1]. This may have been related to urbanization, as many small town and rural development projects were carried out during that period, including infrastructure projects and rural community water supply schemes, involving gravity-based water systems, boreholes, and wells, etc. These water schemes resulted in shared water collection points in the community; therefore, refillable domestic water containers are commonly used in households in Lao PDR. However, the WHO reported that 80% of the refillable water storage containers used in Lao PDR and Cambodia, such as jars, drums, and concrete tanks, act as mosquito breeding sites, where the *Aedes* mosquitoes that carry DENV can lay their eggs [3].

Humans that are bitten by DENV-infected female *Aedes* mosquitoes may develop clinically symptomatic or asymptomatic infections. However, humans with asymptomatic DENV infections can transmit DENV to mosquitoes [4]. The human immune system mainly consists of innate immune and adaptive immune systems. The innate immune system can quickly destroy invading DENV, but does not provide long-term protection against DENV, while the adaptive immune system provides long-term protection against DENV, particularly immunoglobulin G (IgG), which specific antibody that recognizes DENV, and works together with cytotoxic T cells to kill DENV-infected cells and neutralize the virus [5,6]. IgG antibodies play an important role in preventing various infectious diseases, and their levels increase significantly after antigen stimulation, especially during secondary responses. Therefore, the dengue virus Envelop/Membrane protein-specific IgM/IgG ratio can be used to distinguish primary from secondary DENV infections [7].

Vector-control strategies for preventing DENV infections in DENV-endemic areas are based on controlling mosquito activity. However, no such vector-control activities or tools have been classed as a priority. Therefore, the WHO has suggested that an appropriate insecticide should be used in conjunction with other vector-control strategies. In a previous study, it was reported that in Thailand integrating simple techniques improved the vector control of *Aedes* mosquitoes, and the outcomes of these activities were monitored by performing a seroprevalence analysis based on a commercial (COM) ELISA [8].

A number of immunological tests for DENV infections are now commercially available, the COM ELISA is one, which is used to detect IgM, IgG, and the NS1 glycoprotein. The COM ELISA is designed for diagnosing dengue after onset that widely used for the laboratory confirmation of DENV infections. As is well known, COM ELISAs are based on cut-off value that was computed or analyzed between OD values of positive and negative control as the key to discriminate between positive and negative cases for dengue diagnosis. On the other hand, the cut-off value in each COM ELISA kit is different among suppliers. However, COM ELISAs are also commonly used for epidemiological studies of herd immunity after previous DENV infections. A number of studies of COM ELISA from different manufacturers found that they exhibited varying sensitivity and specificity when used to test the same DENV-infected samples [9,10,11]. The ELISA are also widely used to detect asymptomatic DENV infections or past DENV exposure in epidemiological studies [12,13]. In Lao PDR, most domestic refillable water containers are breeding sites for *Aedes* mosquitoes, and suspected dengue cases are reported every year during the rainy season (July–August). Epidemiological studies of dengue transmission have reported high levels of endemicity in both urban and rural communities in Lao PDR [14,15], therefore, to use COM ELISA for indirect evaluation of mosquito vector-control activity seems not suitable, because most COM ELISA kits designed for qualitative analysis, but is not for quantitative analysis using human serum as samples in endemic area of dengue infection.

Recently, we have reported on an entomological study of DENV mosquito vector control [16], which was coherent with this study as a direct evaluation after used the SumiLarv^TM^ 2MR discs. The SumiLarv^TM^ 2MR disc is a long-lasting matrix release formulation, which contains 2% (*w*/*w*) pyriproxyfen, an insect growth regulator which is used to control *Aedes* mosquito populations in Lao PDR. The result showed that the percentage of water containers infested with *Aedes* mosquito larvae that was primary common vector of DENV transmission to humans was significantly decreased in the treated site, especially in the rainy season, while there was no reduction was seen in the non-intervention site. The SumiLarv^TM^ 2MR discs has been set at 0–0.1 mg/kg bw/day as an acceptable daily intake [17].

In this study, we optimized and used an NS1 ELISA, in which recombinant DENV serotype 2 non structural protein 1 (R-DENV2-NS1) was applied to a polystyrene plate as a coating antigen, to examine anti-DENV IgG antibody levels in human serum samples, in order to indirectly evaluate the efficacy of a larvicide (SumiLarv^TM^ 2MR discs), as follow up analysis in the same study areas with previous entomological study of DENV mosquito vector control as mention above [16].

## 2. Materials and Methods

### 2.1. Village Selection

The villages selected are in a rural area of Thakhek District, Khammouane Province, in the central part of Lao PDR were selected as study sites. PhonNyiaNyai village (17°18′53.69′′ N, 104°54′18.63′′ E), which included 180 households and had 869 inhabitants in 2015, and MuangLatKhuay village (17°17′28.07′′ N, 104°54′0.82′′ E), which included 99 households and had 562 inhabitants in 2013, were selected as the non-intervention sites (non-intervention 1 and non-intervention 2, respectively). At the non-intervention sites, water containers were not treated with the larvicidal product. On the other hand, NyangKhao village (17°18′20′′ N, 104°55′20.33′′ E), which consisted of 143 households and had 685 inhabitants in 2015, was selected as the intervention site.

**Figure 1:**
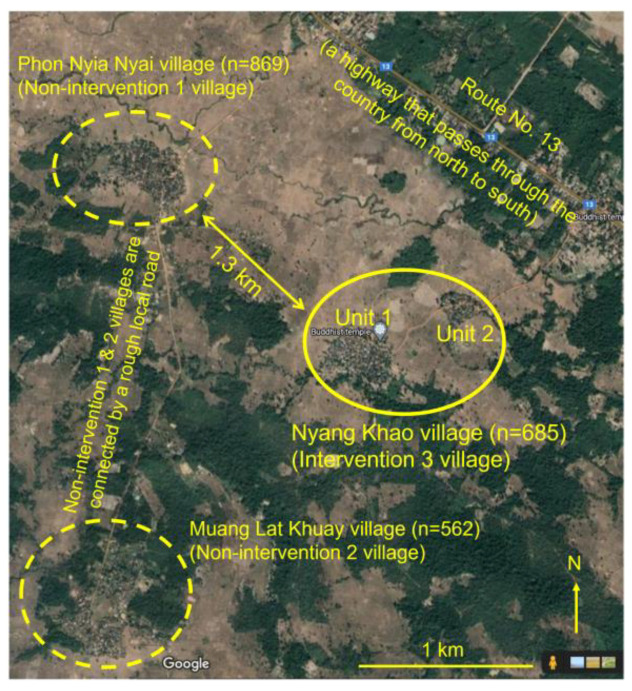
The non-intervention and intervention sites (villages) in Thakhek district, Khammouane Province. The map was modified from Lamaningao et al., 2020 [16]. Used aerial distances measure of Google Maps.

The study site selection was based on the location and village structure indicating that the non-intervention and intervention communities are independent of each other. The non-intervention and the intervention villages were located ≥1.3 km away from each other (Figure 1). The villages were similar in using water conditions that shared public water sources in the village such as dug wells or boreholes, therefore, the households have to use refilled water containers to store enough water for their families, which will be served for the *Aedes* mosquito can lay eggs. Most of the villagers’ daily life based on their household/village’s products, e.g., vegetables, poultries, and others, which they can find in the forest around their villages. In each village, there are retail shops, most villagers do not often go out of their villages. Public transportation is not comfortable from study sites to the center of Thakek town, especially during the rainy season. These villages are occupied by farmers and surrounded by rice fields and spots of the forest. In the non-intervention and intervention villages, there is an elementary school for each village, which the children of ages between 6 and 12 years old use for their education. In these villages, there is a temple of each village, which the villagers use as a community center in the village.

### 2.2. Study Design and Household Selection

As only one intervention site in this field trial study, then the study was designed as a longitudinal study to surveillance of more than three years of observation in both non-intervention and intervention sites, after intervention activity in the intervention site by treatment of refillable domestic water containers by a larvicide. The list of household numbers was created from the village census book of each village. The household numbers were selected from the random number table by software computation such Microsoft Excel spreadsheet software (Microsoft Office 2007, USA). Two of the third household numbers in each village will be asked for voluntary blood sample providing after the random process.

### 2.3. Larvicide

At the intervention site, refillable domestic water containers, as well as the suspected mosquito breeding sites were treated with SumiLarv^TM^ 2MR discs [16]. The discs were changed every 6 months throughout the treatment period (from October 2017 to January 2020; 27 months) as a vector control strategy against *Aedes* mosquitoes. The larvicide exhibits sustained efficacy for at least 6 months in those containers, 1 disc per 40 L of water [18,19].

### 2.4. Serum Samples

The blood samples were collected in two periods after declared the approval permission from the local/institutional Ethic Committee to participants and as well as, after obtained the informed consent documents that were signed by the participants. The first period was in October 2017 (before the larvicidal treatment at the intervention site; pre-intervention). In this period, blood samples were collected from the non-intervention 1 village (PhonNyiaNyai village) of 45 volunteers (aged 2–11 years, median 7, *n* = 7; and aged 13–76 years, median 43.5, *n* = 38) and the intervention 3 village (NyangKhao village) of 45 volunteers (aged 2–12 years, median 6.5, *n* = 10; and aged 14–80 years, median 36, *n* = 35) and then serum samples were prepared and used to provide baseline data for the study. The second period was after the larvicide had been used at the intervention site for 27 months (between October 2017 and January 2020), i.e., in February 2020 (post-intervention). In this period, 47 blood samples were collected from the non-intervention 2 village (MuangLatKhuay village) of only aged 14–80 years (median 53, *n* = 47) and intervention site (aged 3–11 years, median 6, *n* = 5; and aged 13–80 years, median 47.5, *n* = 42). Regarding the non-intervention sites, after most villagers in the non-intervention 1 village (PhonNyiaNyai village) denied to provide sera in post-intervention that could receive only 11 serum samples then the samples were excluded from this study and a non-intervention 2 village (MuangLatKhuay village) was selected instead. The non-intervention 2 village is a neighbor of the non-intervention 1 village, with a similar of using domestic refilled water containers and other environment after an observation, as well as the village is independent of the intervention village. On the other hand, the non-intervention 1 and non-intervention 2 shared the local road to access the high way No. 13, and this local road is the end route at non-intervention 2 village (Figure 1).

In terms of the blood sample numbers collected in each period, the sample numbers collected in the non-intervention villages will be referred to as the same numbers that could collect from the intervention village and then continue to collect from the non-intervention 1 and non-intervention 2 villages. As two-thirds of the household numbers were random and the household members were asked for blood samples providing. However, approximately one-seconds of random households in each site with roughly one family member per household agreed to provide the blood sample.

The dengue asymptomatic serum samples were collected in two periods, i.e., the pre-intervention period (October 2017) and the post-intervention (February 2020). The serum was separated from the whole blood of volunteers from the non-intervention and intervention sites by subjecting the collected blood samples to centrifugation at 2000 rpm for 5 min at 4 °C. A total of 500 μL of serum was kept at −20 °C before being used for the COM ELISA and NS1 ELISA.

### 2.5. COM ELISA: Anti-DENV IgG antibody detection

The anti-DENV IgG antibody levels in the sera collected from the volunteers from non-intervention and the intervention villages during the pre-intervention and post-intervention periods were determined using a COM ELISA kit (Immunospec Corporation, Livonia, MI, USA), which was used according to the manufacturer’s instructions. The serum samples were used to prepare 21-fold diluted solution, and 100 µL of diluted sera was dispensed into each well. The optical density (OD) values of the wells were read with a microplate reader (Bio-Rad, model 680) at a wavelength of 450 nm within 15 min of the stop solution being added. A 655-nm reference filter was employed.

### 2.6. NS1 ELISA

#### 2.6.1. Optimization

R-DENV2-NS1 was selected for the NS1 ELISA. DENV2-NS1 has a similar amino acid sequence to the DENV1, DENV3, and DENV4 NS1 proteins. In addition, DENV2 was also the predominant serotype in the region during the study period [20]. The R-DENV2-NS1 (R&D Systems Biotech, Minneapolis, MN, USA; catalog number 9439-DG) was diluted with 0.1 M carbonate-bicarbonate (pH 9.6) and used to coat a 96-well polystyrene plate for the NS1 ELISA. Initially, the peptide concentrations of 0.1 µg/well, 0.15 µg/well and 0.2 µg/well were used; incubation times of one hour and overnight; serum primary antibody concentrations of 50-fold, 75-fold, and 100-fold. In addition, secondary antibody concentrations of 1000-fold, 1500-fold, and 2000-fold were evaluated to determine the optimal conditions for the NS1 ELISA in a positive sample serum by COM ELISA. The plates were then washed with 0.05% phosphate-buffered saline (PBS) Tween-20 buffer solution (pH 7.4), before being incubated with 5% bovine serum albumin solution as a blocking reagent at 37 °C for one hour. After the plates were washed, 100 μL of diluted serum in 0.05% PBS Tween-20 buffer solution was added to them, before they were incubated again for one hour at 37 °C. Next, the plates were washed, and 200 μL of the horseradish peroxidase-conjugated polyclonal rabbit anti-human IgG secondary antibody in 0.05% PBS Tween-20 buffer solution (Agilent Technologies, Dako, Glostrup, Denmark) was added. The plates were then incubated for one hour at 37 °C, before being washed. Finally, the color was developed using ABST (KPL ABTS, SeraCare, Milford, CT, USA) as a substrate solution. The OD value of the developed color was measured at 405 nm for 30 min, as recommend in the manufacturer’s instructions.

Moreover, of 6 serum samples that were subjected to COM ELISA to detect anti-DENV IgG antibody from non-intervention and the intervention villages, included a negative sample and 2 positive samples from each village were used to determine in deferent R-DENV2-NS1 peptide concentrations from 0.01 µg/well to 0.4 µg/well. The assay based on the results of the optimization process above that used incubation time for overnight at 4 °C, 100-fold dilution for primary antibody and 1500-fold dilution for secondary antibody.

#### 2.6.2. Inhibitory Assay

In order to assess the specificity of R-DENV2-NS1 antigen to anti-DENV IgG antibody in serum samples of study sites that will not be cross reacted to anti-Japanese encephalitis virus (JEV) and anti-chikungunya virus (CHIKV) IgG antibodies, three inhibitory tests were conducted by following the NS1 ELISA protocol above that used R-DENV2-NS1 peptide of 0.15 µg/well for ELISA plate coating. A positive of DENV serum sample from the COM ELISA was used for the assay; the serum was mixed and incubated for an hour with R-DENV2-NS1 antigen, JEV antigen or CHIKV antigen by different concentrations from 0.003 µg/well to 0.4 µg/well.

#### 2.6.3. Evaluation of the Sera

Based on the results of the optimization protocol above then R-DENV2-NS1 peptide of 0.15 µg/well was used to coat the plates then all sera from non-intervention and intervention sites were assayed to detect OD values of pre-intervention and post-intervention periods as NS1 ELISA.

### 2.7. Flow of the Experiment and Data Analysis

First, the serum samples that were collected from each site during the pre-intervention period (in October 2017) were subjected to the COM ELISA to provide baseline data regarding anti-DENV IgG antibody levels. The positive/high-OD-value samples, as well as the negative samples were used to optimize the dilution of R-DENV2-NS1, as mentioned in the section about the NS1 ELISA. After the optimization procedure, a fixed concentration was used to coat the plates used for the NS1 ELISA. All serum samples collected from each site during the pre-intervention (October 2017) or post-intervention (February 2020) period were evaluated using the NS1 ELISA.

A comparison of the OD values obtained for the serum samples collected from non-intervention 1 or non-intervention 2 and the intervention 3 villages during the pre-intervention period (October 2017) and the post-intervention (February 2020) using the COM ELISA with those obtained using the NS1 ELISA was performed, and correlation coefficients were calculated using Spearman’s correlation coefficient.

In addition, the significance of the differences between the OD values of the non-intervention 1, non-intervention 2 and intervention sites during the pre-intervention and post-intervention periods was analyzed using the Mann-Whitney *U* test. All statistical analyses were performed using GraphPad Prism 8.4.3 (GraphPad Software, San Diego, CA, USA), with *p*-values of <0.05 considered significant.

## 3. Results

### 3.1. COM ELISA

**Figure 2:**
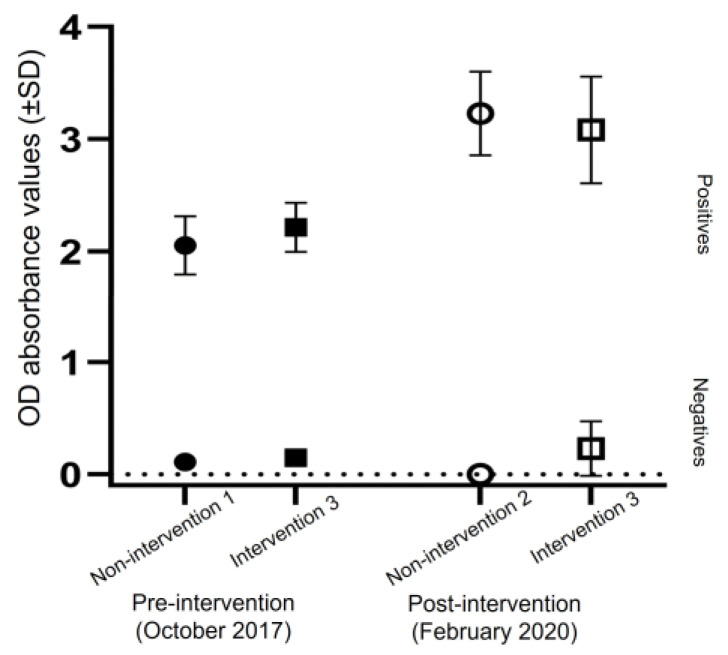
The comparison of the mean OD absorbance values obtained with the COM ELISA.

The COM ELISA were used to detect anti-DENV IgG antibodies in the sera of volunteers from the non-intervention and intervention sites during the pre-intervention (October 2017) and post-intervention (February 2020) periods, and positive samples is 91.1% (*n* = 45); 100% (*n* = 47) for non-intervention sites and 86.67% (*n* = 45); 93.62% (*n* = 47) for intervention site, respectively. In terms of anti-DENV IgG, the mean OD values for positive and negative results obtained for each site using the COM ELISA were high and clearly separated as shown in Figure 2.

### 3.2. NS1 ELISA

#### 3.2.1. Optimization

**Figure 3:**
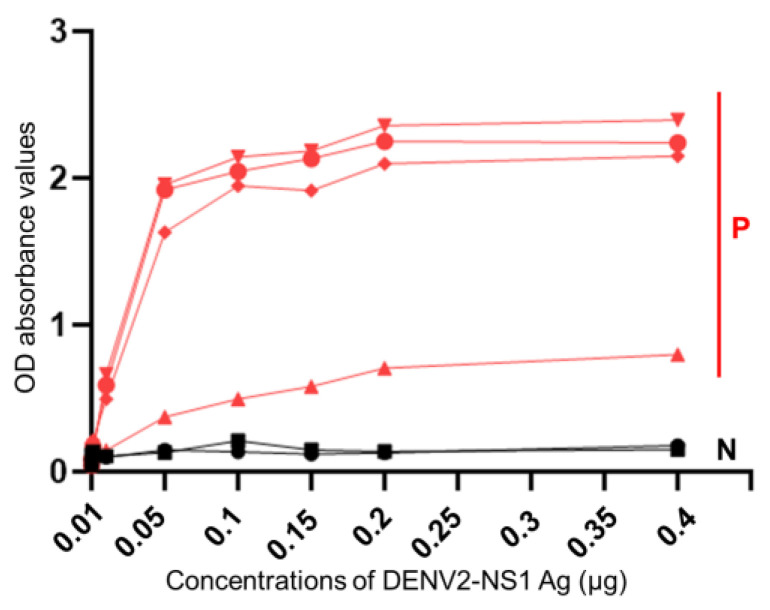
The detection of OD values in deferent R-DENV2-NS1 peptide concentrations of NS1 ELISA on negative and positive samples from COM ELISA. (P) are positive samples, whereas (N) are negative samples.

The protocol of NS1 ELISA test was used to evaluate on different R-DENV2-NS1 peptide concentrations that used to coat the ELISA wells for 2 negative and 4 positive serum samples, the results showed clearly that negative samples by COM ELISA kept low of OD values, while in the positive samples have shown different OD values by slightly increasing followed the low to high of R-DENV2-NS1 peptide concentrations that used coating the ELISA wells (Figure 3).

#### 3.2.2. Correlation Coefficients of OD Values between COM ELISA and NS1 ELISA

**Figure 4:**
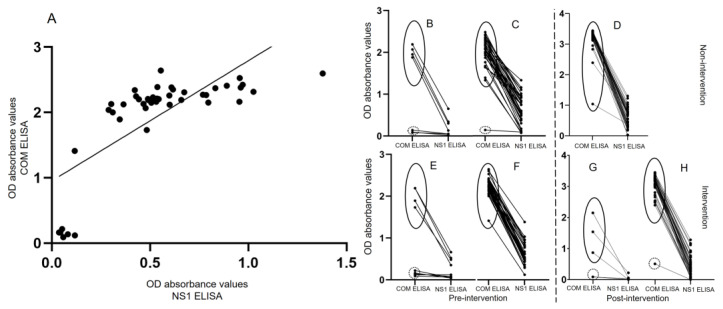
The comparison of the OD absorbance values obtained with the COM ELISA and NS1 ELISA as correlation coefficient measurement for serum samples collected in October 2017 (pre-intervention) and in February 2020 (post-intervention) at the non-intervention (*n* = 45; *n* = 47) and intervention (*n* = 45; *n* = 47) sites, respectively. (**A**) shows correlation OD absorbance values between the COM ELISA and NS1 ELISA is a representative from intervention 3 village in October 2017, while (**B**,**E**,**G**) show individual OD absorbance values of COM ELISA and NS1 ELISA for under 12 of age group, whereas (**C**,**D**,**F**,**H**) show upper 12 of age group of both sites in the pre-intervention and post-intervention periods, respectively. The black circles composed of solid lines indicate high OD values; i.e., positive results for the anti-DENV IgG antibody, according to the COM ELISA. The black circles composed of dashed lines indicate low OD values; i.e., negative results for the anti-DENV IgG antibody, according to the COM ELISA.

The OD values obtained for the pre-intervention (October 2017) and the post-intervention (February 2020) serum samples using the COM ELISA were compared with those obtained using the NS1 ELISA. For the pre-intervention (October 2017) period, the positive correlations between the results obtained with the two ELISAs were observed for non-intervention 1 and intervention 3 (r = 0.6238, 95% CI: 0.4043–0.7753; *n* = 45; r = 0.7423, 95% CI: 0.5738–0.8505; *n* = 45, respectively). For the post-intervention (February 2020) period, the positive correlations were observed for non-intervention 2 and intervention 3 (r = 0.1811, 95% CI: −0.1119–0.4451; *n* = 47; r = 0.562, 95% CI: 0.3277–0.7312; *n* = 47, respectively). At both sites, the samples that were negative according to the COM ELISA also produced lower OD values in the NS1 ELISA (Figure 4).

#### 3.2.3. Inhibitory Assay

**Figure 5:**
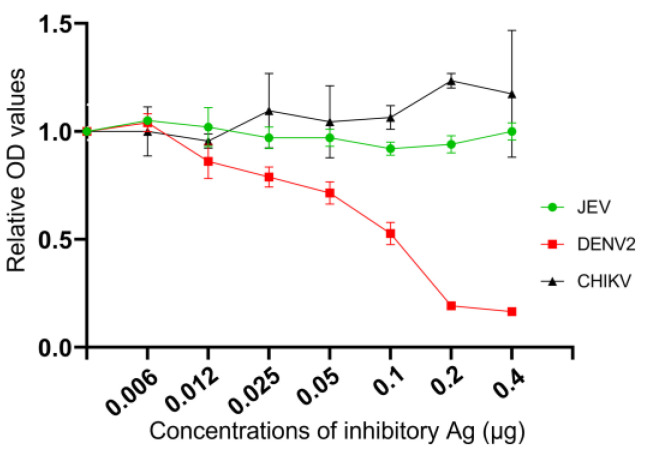
The comparison of the relative OD values obtained using the NS1 ELISA for DENV infected serum sample that was inhibited by R-DENV2-NS1 antigen, JEV antigen and CHIKV antigens in different for cross reaction among anti-DENV IgG, anti-JEV IgG and anti-CHIKV IgG antibodies in the NS1 ELISA. The OD values indicated with triangle markers and error bars were results of serum sample that was absorbed with CHIKV antigen after preincubated, whereas dot markers and error bars were result of serum sample that was absorbed with JEV antigen and while square markers and error bars was absorbed with R-DENV2-NS1 antigen, which were used as primary antibody in inhibitory test.

The infection of a DENV serum sample that was used to inhibit by R-DENV2-NS1 antigen, JEV antigen and CHIKV antigen then subjected to NS1 ELISA test showed on Figure 5. The results showed that the relative OD values which were inhibited by JEV antigen and CHIKV antigen were constraints, while inhibited by R-DENV2-NS1 antigen the relative OD values were decreased from the low to high on different concentrations of inhibitory antigen.

### 3.3. Evaluation of the Sera by NS1 ELISA

**Figure 6:**
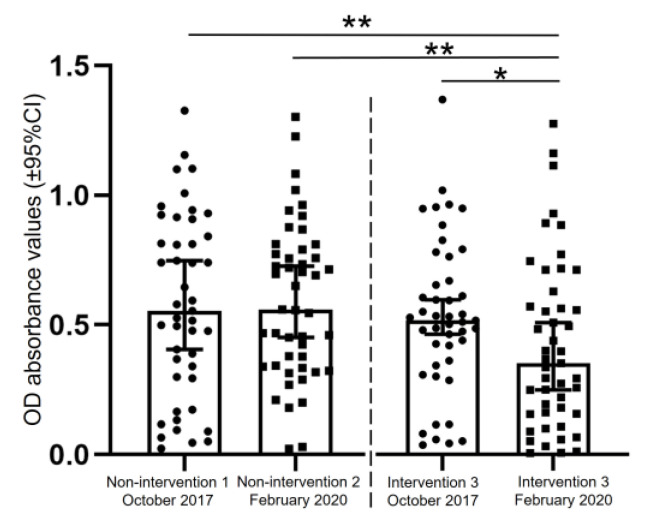
The comparison of the median OD absorbance values obtained using the NS1 ELISA for serum samples collected in October 2017 (pre-intervention) or February 2020 (post-intervention) at the non-intervention or intervention sites. *p*-values were calculated using the Mann-Whitney *U* test (pre-intervention vs. post-intervention: * *p* < 0.05 and ** *p* < 0.01). OD: optical density.

The anti-DENV IgG antibody levels in the serum samples collected from the non-intervention 1 and intervention 3 sites during the pre-intervention (October 2017) and the non-intervention 2 and intervention 3 sites during the post-intervention (February 2020) periods were evaluated using the NS1 ELISA. At the intervention site, it was found that the median OD value (anti-DENV IgG antibody level) decreased significantly (*p* < 0.05) between the pre-intervention (0.52, 95% CI: 0.0365–1.3785; *n* = 45) and post-intervention periods (0.354, 95% CI: 0.0055–1.2845; *n* = 47). Furthermore, the median OD value obtained in the post-intervention period at the intervention site was significantly lower (*p* < 0.01) than those obtained at the non-intervention 1 site during the pre-intervention in 2017 (0.554, 95% CI: 0.0235–1.3265; *n* = 45) and the non-intervention 2 during post-intervention in 2020 (0.559, 95% CI: 0.022–1.303; *n* = 47) periods (Figure 6).

## 4. Discussion

The confirmation for DENV infections in symptomatic cases for laboratory are commonly used two different techniques: (i) A polymerase chain reaction (PCR) technique used to directly detect DENV genome in plasma/sera from patient blood samples at an early stage after infection that was duration of dengue viremia. (ii) The COM ELISA technique used to detect related specific immunoglobulins (IgM and IgG) anti-DENV antibody. In addition, COM ELISA that specifically detect IgG or IgM have been designed in order to allow secondary DENV infections to be distinguished from primary DENV infections [10,21]. In some studies, the frequency of DENV IgG- and/or IgM-seropositivity at study sites was determined using COM ELISA in order to evaluate the effectiveness of dengue vector-control programs [8,22].

Nevertheless, the COM ELISA from different providers or suppliers shown different results on sensitivity and specificity as well. The extent to which serum samples should be diluted varies among COM ELISA kits due to differences in the concentrations of the proteins used to coat polystyrene plates in the assays. On the other hand, COM ELISA OD values do not change in a linear manner depending on the concentration of the coating protein. COM ELISA OD value curves consist of three parts, the baseline, exponential, and plateau sections, which are affected by the concentration of the protein used to coat the polystyrene plate, the concentration of the test samples, and the OD reading time. When the recorded OD values exceed the maximum limit, the curve will plateau. As the demonstration in this study showed that anti-DENV antibody titers were different in sample to sample. However, the OD values tend to increase on each sample and seemed to be plateau curve at high concentration of R-DENV2-NS1 antigen that used to coat ELISA well, the OD value curves were difference among positive samples of themselves on each point, while negative samples from COM ELISA were not (Figure 3). This study was conducted in Thakhek City, Khammouane Province, where dengue is endemic, as the results obtained using the COM ELISA in the current study, as Figure 4 shows clear differences between the individual OD values obtained using the COM ELISA and NS1 ELISA, the COM ELISA showed high OD values in upper 12 years old group. The result indicated that the study subjects had a history of DENV infection in both study sites. We optimized and used R-DENV2-NS1at the predetermined optimal concentration when coating the polystyrene plates in the ELISA, in order to control the number of antibodies in the serum samples and avoid the maximum OD value being exceeded and the ELISA curve plateauing. Moreover, the R-DENV2-NS1 antigen used in this study was not cross reaction with anti-JEV antibody and anti-CHIKV antibody in the DENV infection serum sample as confirmed by the inhibitory test (Figure 5) that used JEV antigen and CHIKV antigen to inhibit anti-DENV IgG in serum sample, the result showed OD values kept in similar level, while inhibited by R-DENV NS1 the OD values slightly decreased.

Our previous entomological study found two *Aedes* species, *Ae. aegypti* and *Ae. albopictus*, which are common vectors that transmit DENV to humans, in non-intervention village (PhonNyiaNyai) and intervention village (NyangKhao) [16]. Therefore, it is likely that the residents of these villages are repeatedly bitten by mosquitoes, and hence, their serum levels of related antibodies, such as anti-DENV IgG antibodies, will be high then indicated that the study areas were endemicity of DENV.

Humans DENV infections can be asymptomatic or symptomatic. In both types of infection, IgG antibodies are responsible for DENV neutralization, and such antibodies persist in asymptomatic DENV-infected individuals for a few years, offering temporary cross-DENV-serotype protection [23] and longer protection for several years in a symptomatic group that re-infected the same DENV serotype [24]. In the current study, we assessed the effectiveness of vector-control activity against *Aedes* mosquitoes (treating refillable water storage containers with SumiLarv^TM^ 2MR discs) in a rural area by analyzing anti-DENV IgG antibody levels at non-intervention and intervention sites during periods when dengue was endemic in Thakhek City, Khammouane Province, using an NS1 ELISA. As a result that used sera to subject on NS1 ELISA, it was found that the median anti-DENV IgG antibody-related OD values obtained at the non-intervention sites during the pre-intervention and post-intervention periods were not significantly different, and at the intervention site the median anti-DENV IgG antibody-related OD value obtained during the pre-intervention period was significantly higher than that obtained during the post-intervention period (Figure 6). An entomological analysis of the efficacy of SumiLarv^TM^ 2MR disc-based vector control targeting *Aedes* mosquitoes conducted in our previous study showed that in the intervention village the prevalence rates of containers infested with *Ae. aegypti* larvae was significantly lower in the post-intervention period than in the pre-intervention period, but in the non-intervention village no reduction in the frequency of *Ae. aegypti* larvae was seen in the post-intervention period compared with the pre-intervention period [16].

We conducted a pilot of a field trial study in a rural area in a south-central part of Lao PDR for assessment on the efficacy of SumiLarv^TM^ 2MR discs, which is a new larvicidal formulation for mosquito vector control, especially against *Aedes* spp. populations. The SumiLarv^TM^ 2MR disc is containing 2% (*w*/*w*) pyriproxyfen and slowly secreted into water with long-lasting effectiveness at least 6 months when added to refillable water containers. After 27 months of larvicidal treatment then we followed to examine sera of villagers for anti-DENV IgG antibody titer by NS1 ELISA test in non-intervention and intervention areas using the recombinant peptide synthesis of DENV2-NS1 coated on ELISA polystyrene plate. The OD absorbance values from NS1 ELISA were analyzed as in dengue endemic area. In the present study, the sample size was small. Therefore, it was not possible to analyze other related factors, such as demographic characteristics. Moreover, we could not continuously distribute the SumiLarv^TM^ 2MR discs and monitor the study site residents for anti-DENV IgG antibodies due to the COVID-19 pandemic, then cannot implement this study as the plan was designed for a longitudinal study, but it is known that temporary cross-DENV-serotype protection persists for a few years, at least two years. Therefore, to gain a clear understanding of the issues examined in this study it would be necessary to conduct further studies involving a greater number of villages, in which refillable water containers continued to be treated and started to monitor for anti-DENV IgG antibody levels for 1 to 2 years more after intervention for 2 years. More issues must be taken into consideration as important points, such as the ones we faced with the residents in first non-intervention site who denied providing sera during the post-intervention period (February 2020). The second non-intervention site was observed and selected in regards to this study.

## 5. Conclusions

Based on the OD values obtained using the NS1 ELISA at the intervention site, the present study indicated that treating refillable water storage containers in rural areas with SumiLarv^TM^ 2MR discs helped to protect residents from *Aedes* mosquito bites, and hence, DENV infections. This was supported by our previous entomological study, which showed that the use of SumiLarv^TM^2MR discs reduced the frequency of such mosquitoes at the intervention site [16]. Taking these findings together suggests that performing a combination of entomological and serological studies can provide information about temporary changes in mosquito populations and DENV transmission. This is especially true when the NS1 ELISA is used instead of a COM ELISA, as this helps to avoid the maximum OD values being exceeded when ELISA targeting anti-DENV IgG antibodies are conducted in areas in which dengue is endemic.
